# Profiling soil microbial communities with next-generation sequencing: the influence of DNA kit selection and technician technical expertise

**DOI:** 10.7717/peerj.4178

**Published:** 2017-12-19

**Authors:** Taha Soliman, Sung-Yin Yang, Tomoko Yamazaki, Holger Jenke-Kodama

**Affiliations:** 1 Microbiology and Biochemistry of Secondary Metabolites Unit, Okinawa Institute of Science and Technology Graduate University, Onna, Okinawa, Japan; 2 National Institute of Oceanography and Fisheries, Cairo, Egypt; 3 Biodiversity Research Center, Academia Sinica, Taipei, Taiwan

**Keywords:** Soil, Microbes, DNA extraction, Commercial kits, Amplicon

## Abstract

Structure and diversity of microbial communities are an important research topic in biology, since microbes play essential roles in the ecology of various environments. Different DNA isolation protocols can lead to data bias and can affect results of next-generation sequencing. To evaluate the impact of protocols for DNA isolation from soil samples and also the influence of individual handling of samples, we compared results obtained by two researchers (R and T) using two different DNA extraction kits: (1) MO BIO PowerSoil^®^ DNA Isolation kit (MO_R and MO_T) and (2) NucleoSpin^®^ Soil kit (MN_R and MN_T). Samples were collected from six different sites on Okinawa Island, Japan. For all sites, differences in the results of microbial composition analyses (bacteria, archaea, fungi, and other eukaryotes), obtained by the two researchers using the two kits, were analyzed. For both researchers, the MN kit gave significantly higher yields of genomic DNA at all sites compared to the MO kit (ANOVA; *P* < 0.006). In addition, operational taxonomic units for some phyla and classes were missed in some cases: Micrarchaea were detected only in the MN_T and MO_R analyses; the bacterial phylum Armatimonadetes was detected only in MO_R and MO_T; and WIM5 of the phylum Amoebozoa of eukaryotes was found only in the MO_T analysis. Our results suggest the possibility of handling bias; therefore, it is crucial that replicated DNA extraction be performed by at least two technicians for thorough microbial analyses and to obtain accurate estimates of microbial diversity.

## Introduction

Determining microbial community structures of environmental samples by means of amplicon next-generation sequencing (NGS) is an important technique in fields such as agriculture, ecology, and human health. Deep sequencing and the capacity to sequence multiple samples make metagenomic sequencing technologies very attractive for exploring microbial species diversity (Hamady et al., 2008; Pinto & Raskin, 2012; Sogin et al., 2006). However, for all NGS approaches, the first crucial step is the isolation of DNA, since any bias introduced in this step will affect the final results, although additional biases can also be introduced subsequently by different sequencing protocols, databases, and data analysis using different algorithms.

Microbial communities in soil participate in diverse ecological interactions between organisms and in biogeochemical processes of nutrient mobilization, decomposition, and gas fluxes (Urbanova, Picek & Barta, 2011). Therefore, metagenomic studies of soil communities are very important to understand these processes. However, compared to aquatic environments, DNA isolation from soil is particularly challenging due to its physicochemical and biological properties, as well as the presence of compounds that inhibit the polymerase chain reaction (Hata et al., 2011; Iker et al., 2013). Three factors need to be considered for a full metagenomic analysis of soils: soil sampling, DNA extraction from microbes in the soil, and data analysis (Bakken, 1985; Lombard et al., 2011). In principle, there are two approaches to DNA isolation. The indirect method first isolates the microorganisms and in the next step, DNA is extracted from the isolates. In the direct method, DNA extraction is conducted without prior isolation of the target organisms. Direct DNA extraction from soils is faster and more accurate than indirect extraction (Knauth, Schmidt & Tippkotter, 2013); therefore, it is now used exclusively. Further improvements of current techniques are important for at least two reasons. First, metagenomics-based community studies must be reproducible within the same laboratory and between different laboratories in order for results to be comparable. Second, even small differences in community composition need to be reproducible, because many bacterial, archaeal, fungal, and other eukaryotic species have yet to be discovered (Taberlet et al., 2012). Hence, bias resulting from DNA isolation must be minimized.

Several studies on this topic have been published recently. Most have analyzed only the quantity and quality of the DNA isolated by various methods (Dineen et al., 2010; Knauth, Schmidt & Tippkotter, 2013; Mahmoudi, Slater & Fulthorpe, 2011; Tanase et al., 2015). Two studies demonstrated that different isolation methods and the use of different commercial kits can influence sequencing results and community analysis, but they focused on bacterial 16S rRNA genes (Bag et al., 2016; Zielińska et al., 2017). We have considerably extended those investigations by assessing not only the quality and quantity of the isolated DNA but also the sequencing outcome and the results of the final bioinformatics analysis of community structure. Furthermore, we have analyzed not only bacterial communities, but also archaea, fungi, and other eukaryotes.

This study evaluated the effectiveness of two commercial DNA isolation kits (MO BIO PowerSoil^®^ DNA and NucleoSpin^®^ Soil) and also variation in results attributable to skill level differences among technicians (R and T). These factors were evaluated to identify potential bias resulting from different kits and their handling, in order to optimize protocols for analysis of soil microbial communities.

## Materials and Methods

### Sampling and DNA extraction

Soil samples were collected from six locations (Masoho (A), Manzamo_1 (B), Manzamo_2 (C), Iriomote (D), Haemidanohama (E) and Kohamajima (F)) in Okinawa Prefecture, Japan (Table 1). Each dry soil sample was mixed well and frozen in sterilized Falcon tubes at −20 °C until use. Two researchers (R and T) independently extracted total DNA in triplicate from soil samples using commercially available MO BIO PowerSoil^®^ DNA Isolation (MO BIO Laboratories, Carlsbad, CA, USA) and NucleoSpin^®^ Soil (Macherey-Nagel, Düren, Germany) kits. Researchers R and T both handled all samples in the same way at the same time. For each sample, 0.25 g of soil were used as starting material. All steps of DNA isolation were conducted according to the respective manufacturer’s protocols. Of the two buffers supplied with the MN kit, we used buffer SL1 with the enhancer for DNA isolation from all MN samples because it consistently yielded the best DNA extraction results. Detailed protocols for the two kits are available online at https://mobio.com/media/wysiwyg/pdfs/protocols/12888.pdf and http://www.mn-net.com/Portals/8/attachments/Redakteure_Bio/Protocols/Genomic%20DNA/UM_gDNASoil.pdf, respectively. Both researchers had equal and ample experience with DNA extraction methods and used the same equipment for all steps. DNA concentration and purity of all samples were determined using a Nanodrop spectrophotometer ND 2000 (Nano-Drop Technologies, Wilmington, DE, USA). Whereas DNA extraction experiments were conducted independently by two researchers, all other steps, such as PCR amplification, purification of PCR products, library preparation, and sequencing, were conducted by only one researcher so as to avoid additional variation in the other steps. Triplicate total DNA samples were barcoded, pooled, and mixed well in one tube.

**Table 1:** Average DNA concentrations and purities (A260/280) when the same samples were prepared by two researchers (R and T) using two different kits (MO and MN).

Locations/Kits	Latitude and longitude	MO_R		MO_T		MN_R		MN_T	
		Conc. (ng/µl)	A260/280	Conc. (ng/µl)	A260/280	Conc. (ng/µl)	A260/280	Conc. (ng/µl)	A260/280
Masoho (A)	26°29′58.1″N–127°51′13.9″E	42	1.81	13.60	1.85	106	1.81	77.73	1.77
Manzamo_1 (B)	26°30′13.8″N–127°50′56.0″E	29	1.78	11.97	1.88	27	1.74	61.00	1.78
Manzamo_2 (C)	26°30′09.9″N–127°50′57.7″E	131	1.83	66.83	1.82	206	1.87	170.17	1.82
Iriomote (D)	24°20′29.8″N–123°48′59.7″E	17	1.78	7.27	1.79	4	1.40	32.37	1.50
Haemidanohama (E)	24°16′28.0″N–123°49′49.7″E	54	1.79	37.60	1.78	122	1.82	124.03	1.81
Kohamajima (D)	24°20′16.2″N–123°58′41.4″E	39	1.85	31.93	1.83	136	1.80	167.53	1.83

### PCR amplifications and sequences

PCR amplifications employed primer sets (attached with Illumina flow cell adapter) that targeted the 16S rRNA gene of bacteria and archaea, an internal transcribed spacer (ITS) region of fungi, and the 18S rRNA gene of other eukaryotes (Table 2). PCR amplification was carried out in a total volume of 20 µL containing 40 ng (10 ng/µL) microbial template genomic DNA, 0.6 µL (10 µM) each of forward and reverse primers, 4.8 µL PCR-grade water and 10 µL 2 × KAPA HiFi HotStart ReadyMix (Kapa Biosystems, Boston, MA, USA). PCR conditions were as follows: 95 °C for 5 min (initial denaturing step), 30 cycles of 20 s at 98 °C, 20 s at 58 °C, and 30 s at 72 °C, followed by a final extension step at 72 °C for 5 min. Amplicons were quality-tested and size-selected using gel electrophoresis (1.2% (w/v) agarose concentration and 1× TAE run buffer). All PCR was conducted after pooling triplicate samples of total DNA isolates. PCR products were cleaned-up using AMPure XP beads (Agencourt^®^ AMPure^®^ XP kit; Beckman Coulter, Brea, CA, USA) according to the Illumina MiSeq protocol for amplicon preparation. The following steps of library preparation and sequencing were performed by the DNA sequencing section of the Okinawa Institute of Science and Technology (OIST) Graduate University. Sequencing was done on an Illumina MiSeq using MiSeq Reagent Kit V3.

**Table 2:** Next-generation primers used for PCR amplification of samples of soil microbial communities.

Marker	Size	Primer’s name	Sequence	Reference
16S rRNA (bacteria)	460 bp	Bakt_341F	5′-CCTACGGGNGGCWGCAG-3′	Herlemann et al. (2011)
		Bakt_805R	5′-GACTACHVGGGTATCTAATCC-3′	
16S rRNA (archaea)	570 bp	340F	5′-CCCTAYGGGGYGCASCAG-3′	Gantner et al. (2011)
		915R	5′-GTGCTCCCCCGCCAATTCCT-3′	Stahl & Amann (1991)
ITS (fungi)	330 bp	ITS3	5′-GCATCGATGAAGAACGCAGC-3′	White et al. (1990)
		ITS4	5′-TCCTCCGCTTATTGATATGC-3′	
18S rRNA (other eukaryotes)	165 bp	1380F	5′-CCCTGCCHTTTGTACACAC-3′	Amaral-Zettler et al. (2009)
		1510R	5′-CCTTCYGCAGGTTCACCTAC-3′	

### Data analyses

Analysis of variance (ANOVA) was performed using IBM SPSS v21.0.0, with a significance level of *P* < 0.05 for differences in DNA concentrations and purities derived from the two kits (MO & MN) and two researchers (R & T). We created four groups (MNR, MNT, MOR, MOT) of raw read sequences for the ANOVA test. We used FastQC v0.11.4 (Andrews, 2010) to assess the quality of raw fastq data files produced by the MiSeq. High-throughput sequences were imported into CLC Genomics Workbench v8.5.1 (QIAGEN, Aarhus A/S, http://www.clcbio.com) according to quality scores of Illumina pipeline 1.8. In order to achieve the highest quality sequences for clustering, paired reads were merged in CLC microbial genomics module v1.1 using default settings (mismatch cost = 1; minimum score = 40; Gap cost = 4 and maximum unaligned end mismatch = 5). Primer sequences were trimmed from merged reads using default parameters (trim using quality scores = 0.05 and trim ambiguous nucleotides = 2), and samples were filtered according to the number of reads. Sequences were clustered and chimeric sequences detected using CLC microbial genomics module v1.1 at a level of similarity 97% of operational taxonomic unit (OTU). Reference OTU data used in the present study were downloaded from the Greengenes database (DeSantis et al., 2006) for 16S rRNA (bacteria and archaea), the Unite database (Koljalg et al., 2013) for ITS (fungi), and the Silva database (Quast et al., 2013) for 18S rRNA (other eukaryotes). The Permutational Multivariate Analysis of Variance (PERMANOVA) test was conducted using CLC Genomics Workbench version 9.0.1 between two kits (MO & MN) and two researchers (R & T) based on Bray–Curtis distance among all taxonomic levels of each community. Alpha rarefaction curve and principle component analysis (PCA) plots were generated among samples using CLC Microbial Genomics Module v1.1. Raw sequences data were submitted to GenBank under accession numbers SRR5286108–SRR5286131.

## Results

For all locations except B and D, both researchers obtained higher DNA yields with the MN kit than with the MO kit (ANOVA, *p* < 0.005) (Table 1). The amount of DNA extracted by researcher R was greater than that extracted by researcher T for all samples using the MO kit (Table 1). Furthermore, the MN kit showed variation in DNA concentration between researchers R and T among samples. Researcher R obtained greater DNA yields from locations A, C, E, and F, whereas researcher T obtained higher yields from locations B and D (Table 1), but these differences were not significant (ANOVA, *p* < 0.50). DNA quality, as judged by the 260/280 nm absorption ratio showed relatively small and insignificant differences between kits (MN and MO) (ANOVA, *p* < 0.50) and between researchers (R and T) (ANOVA, *p* < 0.50) for all sample locations (Table 1). Differences in the number of final sequences read among archaeal sequences were significant (ANOVA, *p* < 0.005) between researchers R and T, but insignificant regarding the two kits (MN and MO) (ANOVA, *p* < 0.50).

In most cases, DNA samples extracted by researcher T produced fewer sequence reads than those by researcher R for both kits across all microbial communities (ANOVA, *p* < 0.05) (bacteria: *p* < 0.005; archaea: *p* < 0.005; fungi: *p* < 0.50; other eukaryotes: *p* < 0.005) (Table 3).

**Table 3:** Number of raw and final sequence reads and number of OTUs produced by Illumina-Miseq for each sample from four microbial communities.

Sample ID	Bacteria			Archaea			Fungi			Other eukaryotes		
	Raw reads	Final reads	OTUs	Raw reads	Final reads	OTUs	Raw reads	Final reads	OTUs	Raw reads	Final reads	OTUs
MNR_A	58,682	9,529	3,200	74,411	67,635	820	194,886	80,773	1,428	105,613	91,177	3,854
MNT_A	32,719	10,199	3,002	36,113	31,439	613	148,000	57,039	1,050	73,392	61,638	2,376
MOR_A	63,502	11,992	3,561	48,996	44,585	861	220,934	72,926	1,189	102,868	89,638	3,531
MOT_A	58,667	7,399	2,118	48,851	39,483	920	159,450	38,309	705	111,250	101,169	3,547
MNR_B	58,981	11,050	2,798	45,601	41,552	526	149,748	62,313	795	104,215	89,987	2,406
MNT_B	33,022	8,516	2,633	37,514	32,605	559	118,056	51,504	845	63,742	51,879	2,203
MOR_B	51,298	8,899	2,606	47,424	43,391	585	159,524	48,806	640	120,285	104,984	2,677
MOT_B	42,661	3,388	1,532	43,081	34,630	733	166,698	32,971	773	105,622	96,722	2,335
MNR_C	44,271	8,415	2,504	46,674	42,605	594	130,274	49,954	1,389	102,243	87,014	3,731
MNT_C	35,861	9,623	2,793	38,853	33,963	541	135,866	83,035	1,585	64,814	51,692	3,332
MOR_C	50,662	8,958	2,566	58,122	53,017	768	160,004	71,424	1,362	104,655	88,374	3,713
MOT_C	46,294	3,587	1,547	41,182	33,899	761	169,140	40,407	1,339	99,057	66,794	2,744
MNR_D	52,920	8,459	2,588	53,194	46,899	747	528,544	217,566	1,467	210,710	176,991	4,658
MNT_D	37,575	12,216	3,418	30,354	25,587	552	107,084	49,015	1,027	65,963	52,840	2,562
MOR_D	50,016	8,845	2,733	47,615	40,994	513	355,556	103,806	1,423	138,397	115,253	3,209
MOT_D	40,872	6,922	2,030	28,914	22,761	211	148,950	31,948	762	109,311	99,200	1,931
MNR_E	49,085	9,420	2,519	66,897	59,607	783	108,196	98,669	383	134,242	104,872	2,983
MNT_E	34,320	12,379	2,840	21,081	17,394	348	104,680	45,766	1,135	67,999	52,891	2,400
MOR_E	64,427	25,205	1,091	43,969	39,098	377	98,210	61,619	341	121,541	94,245	2,730
MOT_E	48,553	8,239	2,236	34,350	27,956	432	149,976	47,711	1,476	118,961	107,436	3,099
MNR_F	59,352	10,475	3,161	44,781	40,269	487	96,170	44,836	412	118,343	89,164	2,615
MNT_F	31,802	7,923	2,649	32,555	27,693	656	100,956	98,958	1,071	56,587	44,063	2,169
MOR_F	58,397	8,275	2,714	58,488	52,689	651	108,194	64,033	342	126,114	94,926	2,758
MOT_F	36,092	4,621	1,856	53,440	43,225	907	160,300	32,569	728	95,154	85,242	2,762

**Notes.**

MN NucleoSpin^®^ Soil Kit MO PowerSoil^®^ DNA Isolation Kit R Researcher R T Researcher T A–F samples id

**Figure 1:**
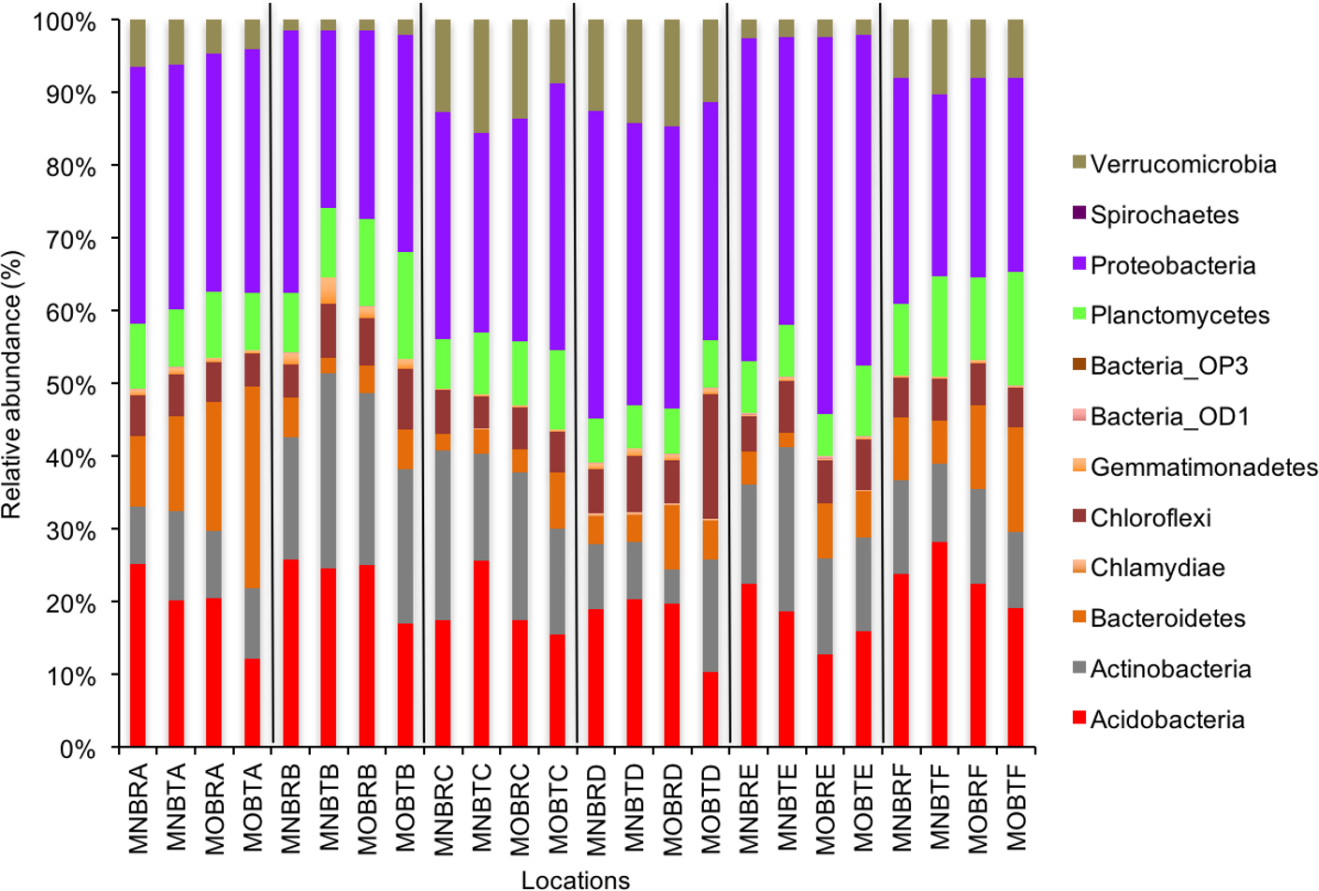
Relative abundance of OTUs of bacterial communities (phyla). MN, NucleoSpin^®^ Soil Kit; MO, PowerSoil^®^ DNA Isolation Kit; R, Researcher R; T, Researcher T; B, Bacteria and A–F sample locations.

**Figure 2:**
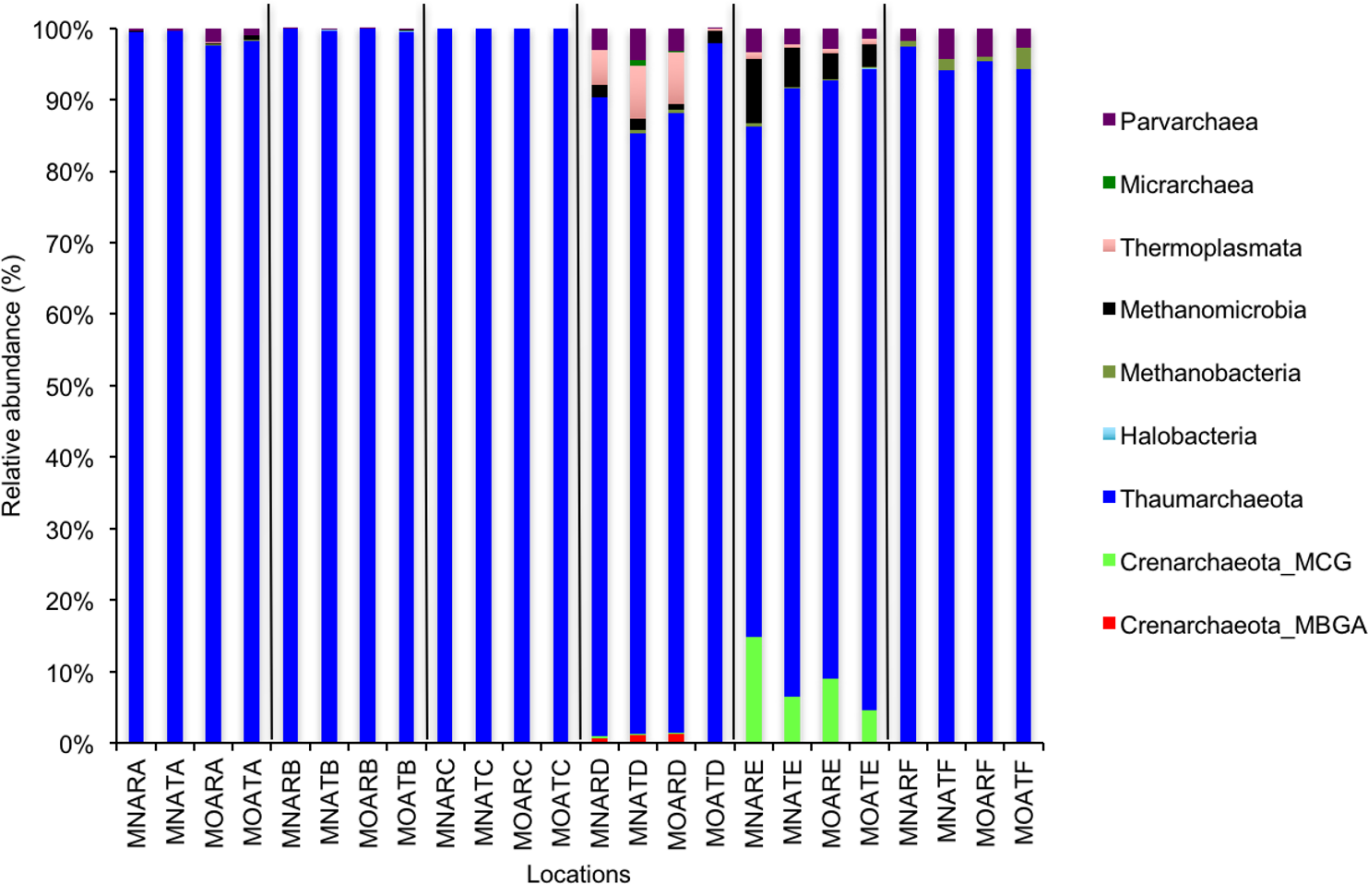
Relative abundance of OTUs for classes of archaeal communities. MN, NucleoSpin^®^ Soil Kit; MO, PowerSoil^®^ DNA Isolation Kit; R, Researcher R; T, Researcher T; A, Archaea A–F sample locations.

**Figure 3:**
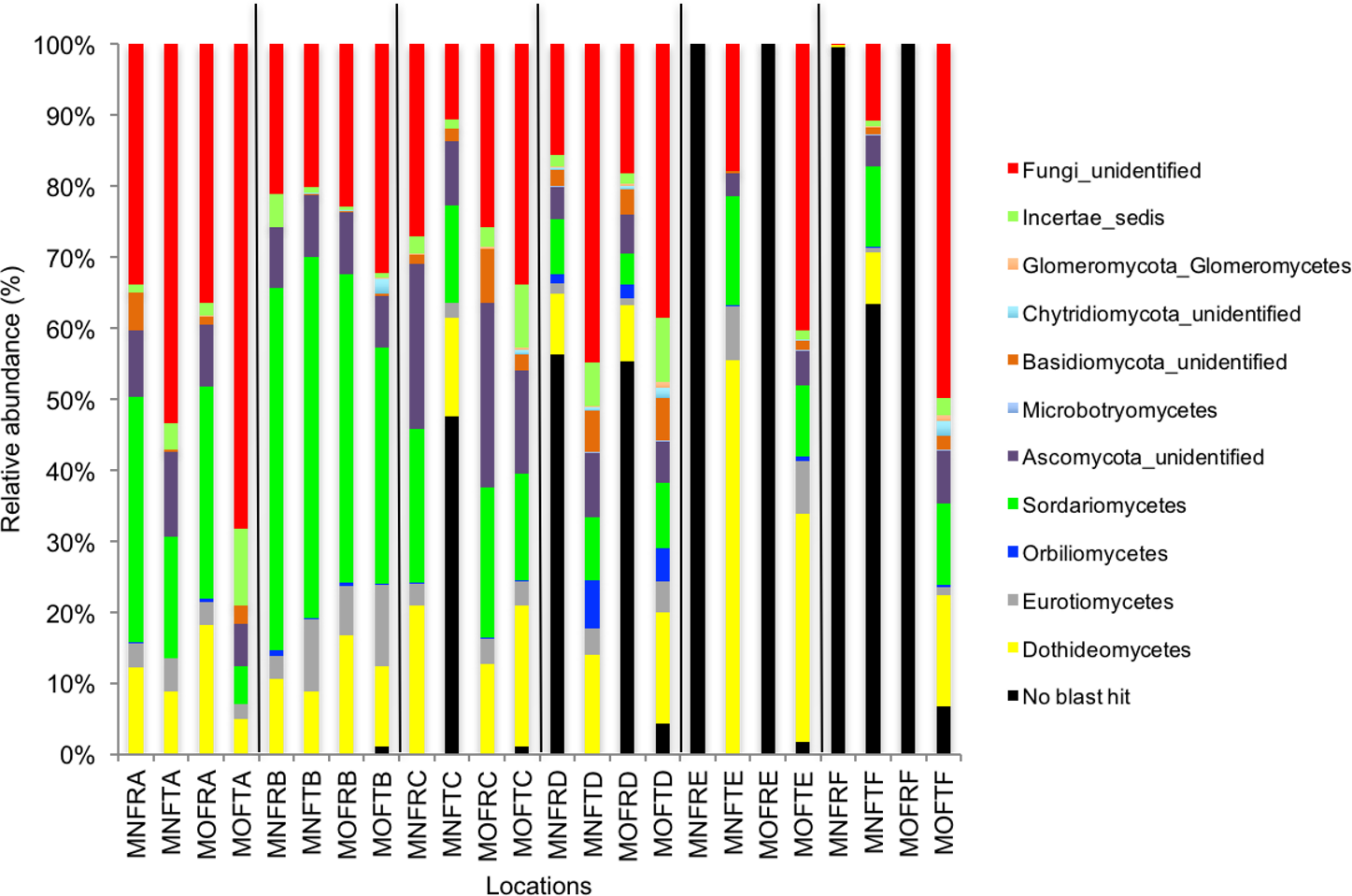
Relative abundance of OTUs of fungal communities (classes). MN, NucleoSpin^®^ Soil Kit; MO, PowerSoil^®^ DNA Isolation Kit; R, Researcher R; T, Researcher T; F, Fungi and A–F sample locations.

**Figure 4:**
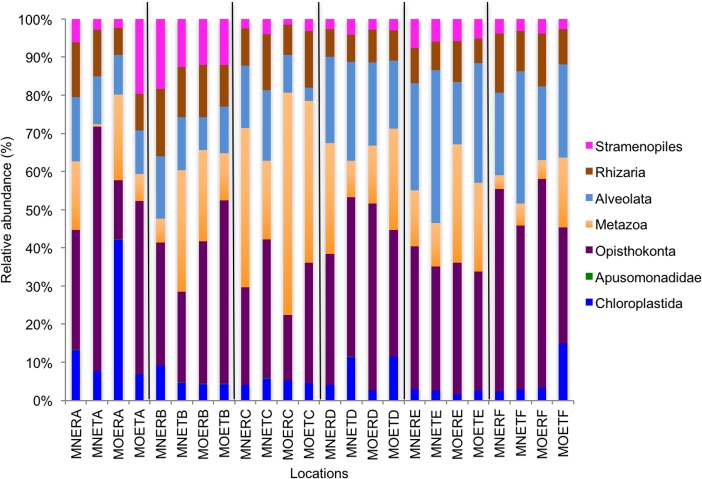
Relative abundance of OTUs among classes of eukaryotic communities. MN, NucleoSpin^®^ Soil Kit; MO, PowerSoil^®^ DNA Isolation Kit; R, Researcher R; T, Researcher T; E, Eukaryotes and A–F sample locations.

We calculated OTUs for all samples defined by 97% sequence identity among the four groups of organisms, i.e., bacteria, archaea, fungi, and other eukaryotes. Taxonomic assignments of bacterial OTUs at the phylum level were dominated by Proteobacteria (32.2%), Acidobacteria (18.9%), Actinobacteria (13.7%), Planctomycetes (8.6%), Bacteroidetes (7.3%), Verrucomicrobia (6.8%), and Chloroflexi (6.0%) across all samples (Fig. 1). Archaeal taxonomic composition at the phylum level included 93.5% Crenarchaeota, 2.4% Euryarchaeota, and 1.5% Parvarchaeota, across all samples. However, the class level composition of archaea was Thaumarchaeota (91.9%), Parvarchaea (1.5%), Crenarchaeota_MCG (1.4%), and Methanomicrobia (1.2%) across all samples (Fig. 2). Calculations of relative abundance showed low differences with both kits and researchers (*p* < 0.05). Among fungi, the dominant phyla were Ascomycota (41.5%), unidentified fungi (22.7%), and Basidiomycota (7.4%) across the various locations (Fig. 3). The most abundant other eukaryotic classes among all locations were Opisthokonta Fungi (33.5%), Opisthokonta Metazoa (18.3%), Alveolata (17.3%), and Rhizaria (9.9%) (Fig. 4).

**Figure 5:**
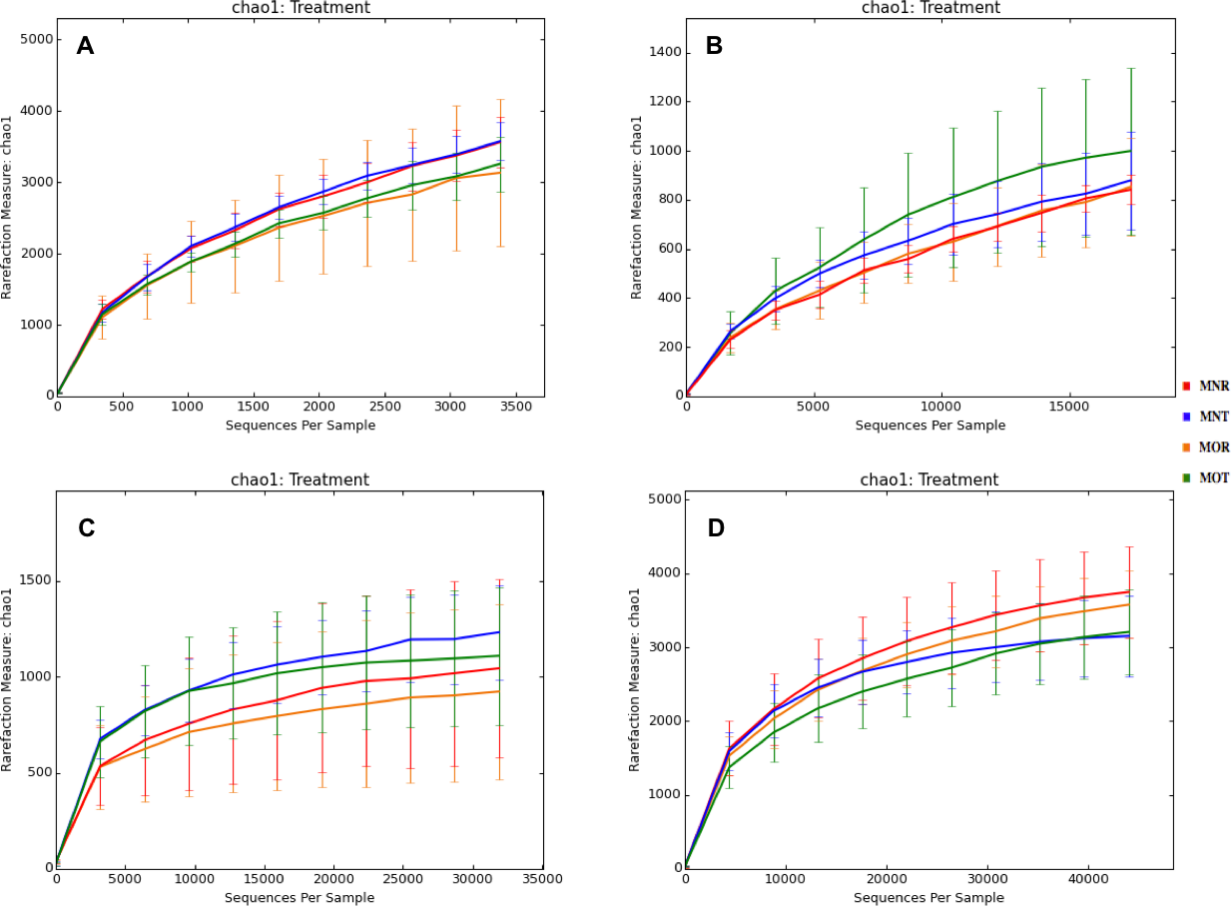
Alpha rarefaction plots between Kits (MN and MO) and researchers (R and T) among four microbial communities. MN, NucleoSpin^®^ Soil Kit; MO, PowerSoil^®^ DNA Isolation Kit; R, researcher R; T, researcher T. (A) bacteria; (B) archaea; (C) fungi; (D) other eukaryotes.

**Figure 6:**
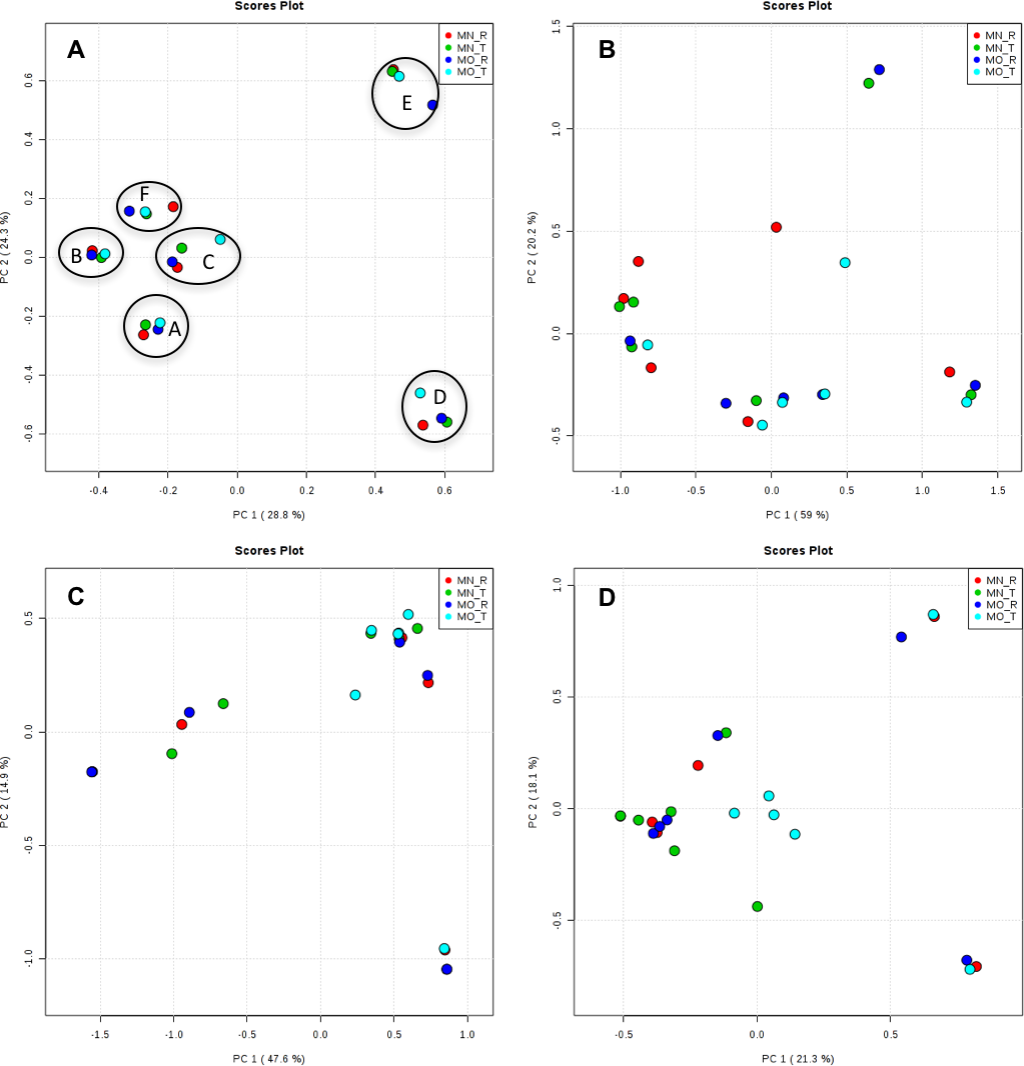
Principal component analysis (PCA) plots of OTUs among kits and researchers. The black circles in bacteria (A) showed one cluster of each sample. A, B, C, D, E, and F; samples name, MN, NucleoSpin^®^ Soil Kit; MO, PowerSoil^®^ DNA Isolation Kit; R, Researcher R, T, Researcher T. (A) bacteria; (B) archaea; (C) fungi; (D) other eukaryotes.

Interestingly, we found a high percentage of no-blast hits for fungal communities for researcher R using both kits at locations D (46.4%), E (99.9%), and F (99.5%), and two locations for researcher T when the MN kit was used (C = 45.5%; F = 51.5%) (Fig. 3). The relative abundance of Micrarchaea was shown only by researcher T (both kits and OTUs of Amoebozoa). WIM5 for eukaryotic communities was also detected by the same researcher (T), but only with the MO kit. In addition, OTUs of Armatimonadetes were detected only by researcher T and only with the MO kit. Overall, the taxonomic diversity among microbial communities were all not statistically significant between two kits (MO & MN) (PERMANOVA, bacteria: *p* = 0.9218, archaea: *p* = 0.8280, fungi: *p* = 0.9703, other eukaryotes: *p* = 0.9638), and between two researchers (R & T) (PERMANOVA, bacteria: *p* = 0.2267, archaea: *p* = 0.2506, fungi: *p* = 0.4494, other eukaryotes: *p* = 0.6757). Alpha rarefaction plots were estimated using the species richness (Chao1), with the number of sequences. In general, the species richness between different researcher and kits were all similar, particularly the archaeal and fungal communities were the same with both kits (MN and MO), but differed by the researcher; whereas for bacterial and eukaryotic communities, the alpha diversity rarefaction curve was relatively similar for both researchers but differed between kits (Fig. 5). Principle component analysis (PCA) showed clusters of each sample for bacteria with slight differences between kits and researcher (Fig. 6A). However, archaea, fungi, and other eukaryotes showed clustering differences among most of the soil samples in the present study (Figs. 6B–6D).

## Discussion

Selection of a DNA extraction kit and protocol is crucial to achieving consistent results for microbial community analysis using NGS technology. Many studies have examined the composition of microbial taxonomic groups in soils and have claimed that unbiased DNA extraction kits and methods are necessary to obtain accurate results (Claassen et al., 2013; Cruaud et al., 2014; Deiner et al., 2015; McOrist, Jackson & Bird, 2002; Tang et al., 2008; Vishnivetskaya et al., 2014). In this study, we investigated the impact of handling methods and DNA extraction kits among four microbial communities (bacteria, archaea, bacteria, fungi, and other eukaryotes). The two DNA kits showed clear differences in DNA yield for both kits (MO and MN) and researchers (R and T). The MN kit produced a higher DNA yield overall. This result may be due to the bead-beating protocol, the type of beads, and differences in the chemical reagents of the two kits. Knauth, Schmidt & Tippkotter (2013) and Finley et al. (2016) reported that for soil, the MN kit yielded more DNA than other kits (FastDNA^®^ SPIN kit (MP Biomedicals, Solon, OH, USA)), the NucleoSpin^®^ soil kit (Macherey-Nagel, Duren, Germany), and the Innu-SPEED soil DNA kit (Analytik Jena AG, Jena, Germany)). In addition, researcher T obtained lower DNA yields than researcher R for most locations using both kits.

We found that the type of kit and handling both affect the DNA yield from soil samples. Some previous studies on soils and feces have shown that the type of DNA isolation kit used significantly affected the results of microbial community analysis and that higher yields of genomic DNA produced a more comprehensive picture of microbial communities (Knauth, Schmidt & Tippkotter, 2013; Claassen et al., 2013; Ariefdjohan, Savaiano & Nakatsu, 2010). In contrast, our finding using the Illumina MiSeq platform showed that the MO kit yielded a greater abundance of OTUs. Mackenzie, Waite & Taylor (2015) reported that the most effective DNA extraction kit for the human gut microbiome is MO, because of the quality of the DNA it produces. Our results differ from those of some previous studies, possibly due to differences between the Denaturing Gradient Gel Electrophoresis (DGGE) and MiSeq techniques (Knauth, Schmidt & Tippkotter, 2013; Claassen et al., 2013; Ariefdjohan et al., 2010). As per DNA isolation protocols, the MN kit has two different spin columns: a red ring spin column to remove inhibitors such as humic substances, and a green ring spin column to wash and bind DNA. So, for both kits, the richness of OTU profiles of microbial communities may differ depending upon the spin column type. Pooling DNA extractions from individual soil samples increased OTU richness (Song et al., 2015). Triplicate DNA extractions using different handling methods for replicates with the same kit have been recommended to avoid biases of NGS analysis and to enhance richness by isolating unique OTUs. Our results with both DNA extraction kits yielded similar DNA purity among samples and relatively similar OTU compositions. However, the OTUs of bacterial phylum Armatimonadetes and WIM5 of eukaryotes were obtained only by MO kit. Therefore, we assume that the MO kit is the most appropriate for DNA extraction from soil.

The DNA extraction protocols influenced the structure of environmental soil microbial communities (Morgan, Darling & Eisen, 2010; Zielińska et al., 2017). Some studies recommended that many DNA extraction kits should test for environmental soil samples in the beginning of the study (Morgan, Darling & Eisen, 2010; Zielińska et al., 2017). However, we assume some laboratories cannot apply many kits for DNA extractions because of funding limitations and other factors. Therefore, our results stated that using different hands in triplicate of the DNA extraction might be one solution to reach a better protocol for soil metagenomics studies.

Community composition data produced in microbial ecology using metagenomics sequencing are not only important for a specific study but are also valuable for meta-analyses that compare results obtained by different research groups. The problem of possible bias in such data introduced by differences in sample handling and methodology was early realized and therefore, several studies analyzed the issue of standardization in microbial ecology (reviewed in Philippot et al., 2012). One needs to distinguish between standardization in the sense of general experimental standards for sample handling and analysis and strictly standardized procedures as they are for instance defined by the International Organization of Standardization (ISO). To achieve the latter, Philippot and colleagues developed and validated a protocol for directly extracting DNA from soil samples (Petric et al., 2011; Philippot et al., 2012), which was accepted by the ISO and is now known as the ISO-11063 method. This standard has been further evaluated in a series of follow-up studies (Plassart et al., 2012; Terrat et al., 2015). Although such strictly defined standards seem to be the most promising way to achieve inter-laboratory comparability of data, there are several issues connected to them. Firstly, it may be necessary to modify an established standard to improve recovery of certain microbial communities and to adjust it to progresses in sequencing technology. For example, one of the follow-up studies regarding the ISO-11063 procedure demonstrated that the protocol gave good results for bacterial soil communities but was less efficient for fungal diversity (Terrat et al., 2015). Therefore, the authors recommended modifying the original procedure by changing the cell lysis step, which is the very first step of all DNA extraction methods. However, if an ISO standard regularly needs modifications, it will weaken its usefulness in comparing data that were collected over longer periods of time. Secondly, it seems unrealistic to expect research groups all over the world to accept a strict standard that does not allow using commercial extraction kits of their own choice thereby also limiting the potential of improving kit efficiency. The most important issue, however, is that even the most strictly defined standard cannot avoid the handling bias. A comprehensive study of inter- and intra-laboratory variations in microbial community analyses demonstrated the possibility of significant differences in the results even when samples were processed in the same laboratory but by different investigators (Pan et al., 2010), which is consistent with our results. This type of bias appears to be independent of the DNA extraction protocol applied. In our opinion, it is in terms of practicability very difficult to introduce truly strict standards that are accepted by the research community. It might be more successful to define a set of standards of good experimental practice. Good practice in microbial community analysis of soils and other environments should include using different hands on triplicates of extracted DNA in order to minimize handling bias. This recommendation is also important when considering the design of any standard protocol in microbial ecology.

## Conclusions

Our findings indicate that the type of DNA isolation kits used and laboratory handling of samples both influence the results of microbial soil community analysis. However, the yield of extracted DNA and the numbers of raw reads sequenced have a significant impact on the number of OTUs across all communities. If the amount of soil sample and initial extraction of DNA yield is not very low, we recommend that researchers should consider that the microbial DNA isolation be done in triplicate by at least two persons to obtain more accurate results when using amplicon sequences (Illumina-MiSeq).

## Additional Information and Declarations

### DNA Deposition

The following information was supplied regarding the deposition of DNA sequences: Raw sequences data were submitted to GenBank under accession numbers SRR5286108–SRR5286131.
